# Karyotypes, male meiosis and comparative FISH mapping of 18S ribosomal DNA and telomeric (TTAGG) _n_ repeat in eight species of true bugs (Hemiptera, Heteroptera)

**DOI:** 10.3897/CompCytogen.v5i4.2307

**Published:** 2011-11-09

**Authors:** S. Grozeva, V.G. Kuznetsova, B.A. Anokhin

**Affiliations:** 1 Institute of Biodiversity and Ecosystem Research, Bulgarian Academy of Sciences, Blvd Tsar Osvoboditel 1, Sofia 1000, Bulgaria; 2 Zoological Institute, Russian Academy of Sciences, Universitetskaya nab. 1, St. Petersburg 199034, Russia

**Keywords:** Karyotypes, meiosis, FISH, 18S rDNA, telomeres, dot-blot, *Deraeocoris*, *Megaloceroea*, *Nabis*, *Cimex*, *Oxycarenus*, *Pyrrhocoris*, *Eurydema*, *Graphosoma*, Heteroptera

## Abstract

Eight species belonging to five true bug families were analyzed using DAPI/CMA_3_-staining and fluorescence *in situ* hybridization (FISH) with telomeric (TTAGG)_n_ and 18S rDNA probes. Standard chromosomal complements are reported for the first time for *Deraeocoris rutilus* (Herrich-Schäffer, 1838) (2n=30+2m+XY) and *Deraeocoris ruber*(Linnaeus, 1758) (2n=30+2m+XY) from the family Miridae. Using FISH, the location of a 18S rDNA cluster was detected in these species and in five more species: *Megaloceroea recticornis* (Geoffroy, 1785) (2n=30+XY) from the Miridae; *Oxycarenus lavaterae* (Fabricius, 1787) (2n=14+2m+XY) from the Lygaeidae s.l.; *Pyrrhocoris apterus* (Linnaeus, 1758) (2n=22+X) from the Pyrrhocoridae; *Eurydema oleracea* (Linnaeus, 1758) (2n=12+XY) and *Graphosoma lineatum* (Linnaeus, 1758) (2n=12+XY) from the Pentatomidae. The species were found to differ with respect to location of a 18S rRNA gene cluster which resides on autosomes in *Oxycarenus lavaterae* and *Pyrrhocoris apterus*, whereas it locates on sex chromosomes in other five species. The 18S rDNA location provides the ﬁrst physical landmark of the genomes of the species studied. The insect consensus telomeric pentanucleotide (TTAGG)_n_ was demonstrated to be absent in all the species studied in this respect, *Deraeocoris rutilus*, *Megaloceroea recticornis*, *Cimex lectularius* Linnaeus, 1758 (Cimicidae), *Eurydema oleracea*, and *Graphosoma lineatum*, supporting the hypothesis that this motif was lost in early evolution of the Heteroptera and secondarily replaced with another motif (yet unknown) or the alternative telomerase-independent mechanisms of telomere maintenance. Dot-blot hybridization analysis of the genomic DNA from *Cimex lectularius*, *Nabis* sp. and *Oxycarenus lavaterae* with (TTAGG)_n_ and six other telomeric probes likewise provided a negative result.

## Introduction

Fluorescence *in situ* hybridization(FISH), established in the 1980s, represents a powerful cytogenetic technique for a visualization of specific DNA sequences onto chromosomes, generating detailed chromosome mapping of eukaryote genomes (Pinkel et al. 1986). Despite the fact that the FISH mapping of insect chromosomes has been under way for a number of years (reviewed by Frydrychová et al. 2004; Vítková et al. 2005), the information of this sort for true bugs is still very scanty and available only for few species studied in respect to telomeric sequences (Okazaki et al. 1993; Sahara et al. 1999) and the location of ribosomal RNA genes (Cattani et al. 2004; Severi-Aguiar et al. 2005, 2006; Papeschi and Bressa 2006; Morielle-Souza and Azeredo-Oliveira 2007; Bressa et al. 2008, 2009; Panzera et al. 2010; Grozeva et al. 2010; Bardella et al. 2010; Poggio et al. 2011).

To fill this gap and learn more about bug genomes, we applied FISH technique with telomeric (TTAGG)_n_ and 18S rDNAprobes to eight species belonging to 7 genera, 5 families and 2 infrforders: *Deraeocoris ruber* (Linnaeus, 1758), *Deraeocoris rutilus* (Herrich-Schäffer, 1838), and *Megaloceroea recticornis* (Geoffroy, 1785) from the family Miridae, *Cimex lectularius* Linnaeus, 1758 from the family Cimicidae (all from the infraorder Cimicomorpha); *Oxycarenus lavaterae* (Fabricius, 1787) from the family Lygaeidae s.l., *Pyrrhocoris apterus* (Linnaeus, 1758) from the family Pyrrhocoridae, *Eurydema oleracea* (Linnaeus, 1758) and *Graphosoma lineatum* (Linnaeus, 1758) from the family Pentatomidae (all from the infraorder Pentatomomorpha). The 18S rDNA location provided the ﬁrst physical landmark of the genomes of the species studied. The species *Deraeocoris ruber* and *Deraeocoris rutilus* were studied here for the first time likewise in terms of their standard chromosomal complements.

In five species, *Megaloceroea recticornis*, *Deraeocoris rutilus*, *Cimex lectularius*, *Eurydema oleracea*, and *Graphosoma lineatum*,we used a (TTAGG)_n_ telomeric probe to justify a hypothesis that this telomeric motif is absent in the true bugs (Frydrychová et al. 2004). The last hypothesis has been so far based only on studies of two species, *Halyomorpha halys* (Stål, 1855) (Okazaki et al. 1993: as *Halyomorpha mista* (Uhler, 1860)) and *Pyrrhocoris apterus* (Sahara et al. 1999) that do not adequately represent the diversity of the Heteroptera.

Additionally, we carried out a dot-blothybridization of the genomic DNA from *Cimex lectularius*, *Nabis* sp. and *Oxycarenus lavaterae* with seven types of telomeric probes, ciliate (TTTTGGGG)_n_ and (TTGGGG)_n_, nematode (TTAGGC)_n_, insect (TTAGG)_n_, shrimp (TAACC)_n_, vertebrate (TTAGGG)_n_, and plant (TTTAGGG)_n_.

## Material and methods

### Insects

Adult males of *Deraeocoris ruber*, *Deraeocoris rutilus*, *Megaloceroea recticornis*, *Nabis* sp., *Cimex lectularius*, *Oxycarenus lavaterae*, *Pyrrhocoris apterus*, *Eurydema oleracea*, and *Graphosoma lineatum* were collected in the vicinities of Plovdiv and Sofia, Bulgaria in 2009-2011 (Table 1). On capture, specimens were immediately fixed in a Carnoy fixative (3 parts of 96% ethanol and 1 part of glacial acetic acid) and stored at 4°C until required.

**Table 1.** Material analyzed

**Infraorder, family, and species**	**Locality in Bulgaria**	**Date of collection**	**Number of specimens analyzed**
**Cimicomorpha**			
**Miridae**			
*Deraeocoris ruber*	Bulgaria, Western Rhodopes Mts., near Kuklen Vill., 42.032990°N, 024.774537°E, 384 m a.s.l.	9.06.2009	2
*Deraeocoris rutilus*	Bulgaria, Western Rhodopes Mts., near Kuklen Vill., 42.032990°N, 024.774537°E, 384 m a.s.l.	8.06.2009	2
*Megaloceroea recticornis*	Bulgaria, Asenovgrad, 42.05977°N, 024.813424°E, 177m a.s.l.	9.06.2009	12
**Nabidae**			
*Nabis* sp.	Bulgaria, Sofia, City Center	15.06.2010	4
**Cimicidae**			
*Cimex lectularius*	Bulgaria, Sofia, Studentski Grad	14.10.2010	3
**Pentatomomorpha**			
**Lygaeidae**			
*Oxycarenus lavaterae*	Bulgaria, Sofia, City Center, on *Tilia* sp.	3.07.2011	4
**Phyrrocoridae**			
*Pyrrhocoris apterus*	Bulgaria, Sofia, City Center, on *Tilia* sp.	3.07.2011	2
**Pentatomidae**			
*Eurydema oleracea*	Bulgaria, Western Rhodopes Mts., near Progled Vill., 41.68067°N, 024,70527°E, 1320 m a.s.l.	9.06.2009	2
*Graphosoma lineatum*	Bulgaria, Thracian Lowland, outflow of Chaya River in Maritsa River, 42.147653°N, 024,880186°E, 152 m a.s.l.	8.06.2009	3

### Preparations

The gonads were dissected out and squashed in a drop of 45% acetic acid. The cover slip was removed using the dry ice. Slides were dehydrated in fresh fixative and air dried. The preparations were first analyzed with a phase contrast microscope at 400x. The best chromosome spreads were used for different staining techniques.

### Fluorochrome banding

To reveal the base composition of C-heterochromatin, staining by GC-specific chromomycin A_3 _(CMA_3_) and AT- specific 4-6-diamidino-2-phenylindole (DAPI) was used according to Schweizer (1976) and Donlon and Magenis (1983) respectively, with some modifications. C-banding pretreatment was first carried out using 0.2 N HCl at room temperature for 30 min, followed by 7-8 min treatment in saturated Ba(OH)_2_ at room temperature and then an incubation in 2xSSC at 60°C for 1 h. Furthermore, the preparation (without Giemsa) were stained first with CMA_3_ (0.4 μg/ml) for 25 min and then with DAPI (0.4 μg/ml) for 5 min. After staining, the preparations were rinsed in the McIlvaine buffer, pH 7 and mounted in an antifade medium (700 μl of glycerol, 300 μl of 10 mM McIlvaine buffer, pH 7, and 10 mg of N-propyl gallate).

### Fluorescence in situ hybridization (FISH). DNA isolation, PCR amplification, probe generation

Genomic DNA from a male of *Pyrrhocoris apterus* (Heteroptera, Pyrrhocoridae) was isolated using a Chelex-100 extracted method. FISH using a 18S rRNA gene probe was carried out on the chromosomes of *Deraeocoris ruber*, *Deraeocoris rutilus*, *Megaloceroea recticornis*, *Oxycarenus lavaterae*, *Pyrrhocoris apterus*, *Eurydema oleracea*, and *Graphosoma lineatum*. FISH using a telomeric (TTAGG)_n_ probe was carried out on the chromosomes of *Deraeocoris rutilus*, *Megaloceroea recticornis*, *Cimex lectularius*, *Eurydema oleracea*,and *Graphosoma lineatum*. The target 18S rDNAgene was PCR amplified (primers presented in Table 2) from the genomic DNA of *Pyrrhocoris apterus*, and labeled by PCR with biotin. Telomere probe (TTAGG)_n_ was PCR amplified and labeled using primers TTAGG_F and TTAGG_R (Table 2) and Rhodamine-5-dUTP (GeneCraft, Germany).

**Table 2.** PCR primers used in present study

**Name**	**Sequence (5‘ – 3‘)**
18S_F	ACAAGGGGCACGGACGTAATCAAC
18S_R	CGATACGCGAAT GGCTCAAT
Eup_F	TTTTGGGGTTTTGGGGTTTTG
Eup_R	CCCCAAAACCCCAAAACCC
Prot_F	TTGGGGTTGGGGTTGGGG
Prot_R	CCCCAACCCCAACCCCAA
Wrm_F	TTAGGCTTAGGCTTAGGCTT
Wrm_R	GCCTAAGCCTAAGCCTAAG
TTAGG_F	TAACCTAACCTAACCTAACCTAA
TTAGG_R	GGTTAGGTTAGGTTAGGTTAGG
Shr_F	TAACCTAACCTAACCTAACCTAA
Shr_R	GGTTAGGTTAGGTTAGGTTAGG
TTAGGG_F	CCCTAACCCTAACCCTAACCCTAACCCTAA
TTAGGG_R	TTAGGGTTAGGGTTAGGGTTAGGGTTAGGG
Plnt_F	TTTAGGGTTTAGGGTTTAGGG
Plnt_R	CCCTAAACCCTAAACCCTAAA

### FISH procedure

*In situ* hybridization was performed as described by Schwarzacher and Heslop-Harrison (2000) with modifications. In each species, one or two FISH preparations were examined. Chromosome preparations weredehydrated through 70/80/96% Ethanol at RT and treated with 100 μg/ml RNaseA (Sigma) for 60 min at 37°C in a humid chamber; washed three times in 2x SSC (5 min each) at RT; dehydrated through 70/80/96% Ethanol at RT; incubated in 5 mg/ml Pepsin in 0.01 N HCl for 15 min at 37°C; washed sequentially in 1x PBS, in PBSx1/0.05M MgCl_2_ for 5 min each, in 1% PFA in PBSx1/0.05M MgCl_2_ for 10 min, in 1x PBS for 5 min, in PBSx1/0.05M MgCl_2_ for 5 min at RT each; dehydrated through 70/80/96% Ethanol at RT or ice cold and finally, dried. After pretreatment, hybridization mixture containing about 100 ng of labeled probe, 50% formamide, 2×SSC, 10% (w/v) dextran sulfate, 1% (w/v) Tween-20 and 10 µg salmon-sperm DNA was added on preparations. Slides were mounted using glass coverslips and rubber cement. The slides were denaturated for 5 min at 75°C. Then the chromosome slides were incubated for 42–44 h at 37°C. Following hybridization, the slides were washed in 2x SSC for 3 min at 45°C, then in 50% formamide in 2xSSC for 10 min at 45°C, two times in 2x SSC (10 min each) at 45°C, blocked in 1.5% (w/v) BSA/4x SSC/0.1% Tween-20 for 30 min at 37° in a humid chamber. 18S rRNAgene probe was detected with 5μg/ml Avidin-Alexa Fluor 488 (Invitrogen). Detection reaction was performed in 1.5 % BSA/ 4x SSC/0.1% Tween-20 for 1 h at 37°C. Slides were washed three times in 4x SSC/0.02% Tween-20 (10 min each) at 45° and dehydrated through 70/80/96% ethanol at RT. Chromosomes were mounted in a mounting-antifade (ProLong Gold antifade reagent with DAPI, Invitrogen) and covered with a glass coverslip.

### Dot-blot analysis

Genomic DNA from *Cimex lectularius*, *Oxycarenus lavaterae* and *Nabis* sp. was isolated using NucleoSpin Tissue Kit (Macherey-Nagel, Germany) or a standard Phenol/Chloroform nucleic acid extraction protocol. Telomere probes of ciliate (TTTTGGGG)_n_ and (TTGGGG)_n_, nematode (TTAGGC)_n_, insect (TTAGG)_n_, shrimp (TAACC)_n_, vertebrate (TTAGGG)_n_ and plant (TTTAGGG)_n_ were PCR amplified using primers labeled with biotin and presented in Table 2.

About 20 ηg of isolated DNA after denaturation was added drop wise to Hybond N+ nylon membranes (Amersham, Biosciences). Hybridizations were carried out over night in hybridization mixture containing about 100-200 ηg of labeled probe, 50% formamide, 4×SSC, 0.5% (w/v) SDS and 10 µg salmon-sperm DNA at 40 °C. Membranes were washed two times in 2x SSC/0.1% SDS (10 min each) at RT and two times in 0.2x SSC/0.1% SDS (10 min each) at RT (10 min each). Detection procedure was performed according to the Biotin Chromogenic Detection Kit protocol (Fermentas).

### Microscopy and imaging

Chromosome preparations were analyzed under a Leica DM 4000B microscope with a 100x objective. Fluorescence images were taken with a Leica DFC 350 FX camera using Leica Application Suite 2.8.1 software with an Image Overlay module. The preparations were stored partly at Institute of Biodiversity and Ecosystem Research, BAS in Sofia and partly at the Zoological Institute, RAS in St Petersburg.

## Results

### Miridae

***Deraeocoris ruber* Linnaeus, 1758,** 2n=30+2m+XY

Fig. 1a–d

**Figure 1a–d.**
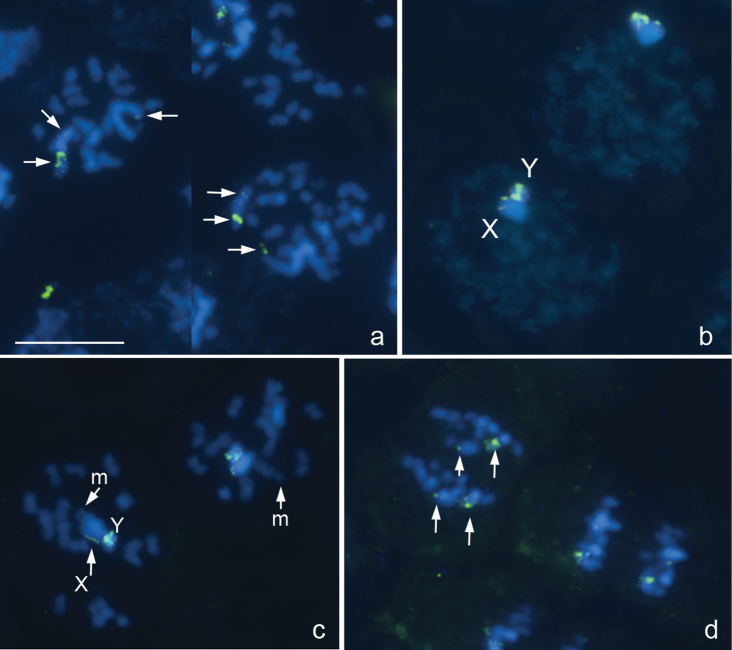
*Deraeocoris ruber*, 2n=30+2m+XY. **a** spermatogonial prometaphase **b** early prophase **c **prometaphase I **d** anaphase II. FISH with an 18S rDNA probe. Arrowed are 18S rDNA clusters **a, d** Bar equals 10 μm.

The karyotype is described here for the first time. There are 16 bivalents, including a pair of very small and negatively heteropycnotic chromosomes taken as a pair of m-chromosomes, and the univalent X and Y chromosomes which are largest and smallest chromosomes in the set, respectively (Fig. 1a–d). Diplotene and diakinesis stages were not detected, and bivalents displayed no chiasmata since meiosis is achiasmate. 18S rRNA genes were mapped on both sex chromosomes, the signals being more intensive on the Y. At spermatogonial metaphases, a number of very small intercalary signals could be in addition seen on the X (Fig. 1a).

***Deraeocoris rutilus* (Herrich-Schäffer, 1838),** 2n=30+2m+XY

Fig. 2a, b

**Figure 2a, b.**
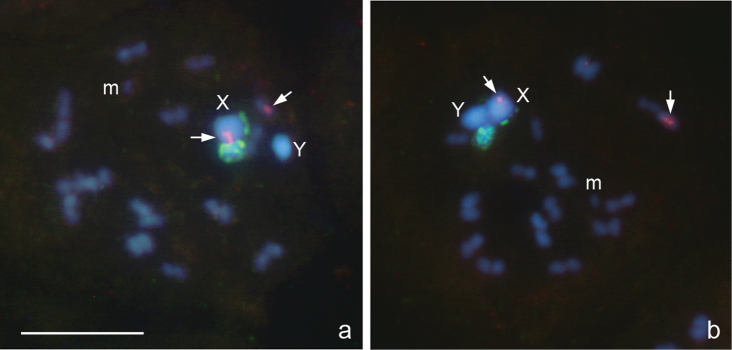
*Deraeocoris rutilus*, 2n=30+2m+XY. **a, b** prometaphase I. FISH with 18S rDNA and (TTAGG)_n_ probes. Arrowed are signals after using a TTAGG-probe. Bar equals 10 μm.

The karyotype is described here for the first time. It is much like that described above for *Deraeocoris ruber*. Likewise, at PMI, there are 16 autosomal bivalents and the univalent X and Y chromosomes. One of the bivalents is very small, negatively heteropycnotic pair of m-chromosomes (Fig. 2a, b). The X is the largest and the Y is one of the smallest chromosomes in the set (excluding the m-chromosomes); autosomal bilvalents constitute a decreasing size raw. Diplotene and diakinesis stages were not observed, and meiosis was considered achiasmate of a collochore type. FISH with an 18S rDNAprobe produced two local signals placed near a telomeric region of the X chromosome and in addition a huge cluster of signals attached to the X; Y chromosome carried no signal (Fig. 2a, b). FISH with a TTAGG probe produced prominent hybridization signals (Fig. 2a, b, arrowed), which were occasionally located on the same chromosomes however most likely did not indicate the telomeres.

***Megaloceroea recticornis* (Geoffroy, 1785),** 2n=30+XY

Fig. 3a–f

**Figure 3a–f.**
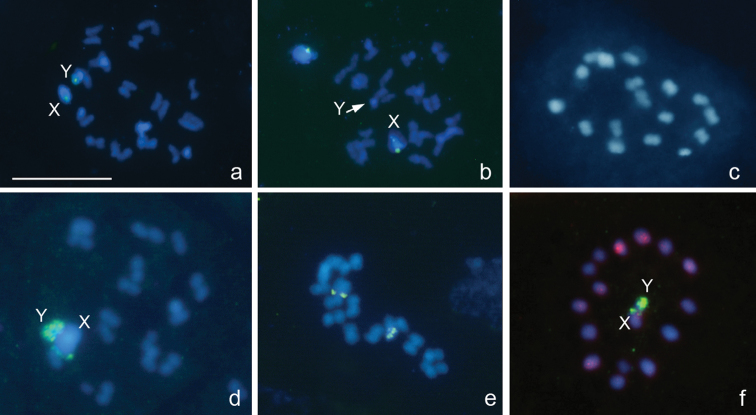
*Megaloceroea recticornis*, 2n=30+XY. **a, b, d** condensation stage; **c, e** prometaphase I; **f** metaphase II. FISH with 18S rDNA **a, b, d–f** and (TTAGG)_n_ **f** probes. Bar equals 10 μm.

From the counts of 23 plates at prometaphase I (PMI), 15 autosomal bivalents and two univalent sex chromosomes, X and Y, were detected suggesting that this species displays 2n=30+XY (Fig. 3a-c) in contrast to 2n=32+XY previously reported for this species in England (Leston 1957). The X is fairly large whereas the Y is one of the smallest chromosomes in the set, and the autosomal bilvalents constitute a decreasing size raw. There are no visible constrictions in the chromosomes, since they are holokinetic. During meiotic prophase, the diplotene and diakinesis stages escaped detection. At condensation stage, the bivalents showed no chiasmata however homologues were connected with each other by tenacious thread-like structures, at least, at one site, and the telomeric regions pushed off from each other (Fig. 3a, b). Taken together, the observations of meiosis suggest this species to display the achiasmate meiosis of a collochore type. Fluorescence *in situ* hybridization with a (TTAGG)_n_ probe did not reveal positive signals on the telomeres although occasionally gave rise to variable interstitial hybridization signals, sometimes quite bright, on separate chromosomes (Fig. 3f). Major ribosomal DNA cistrons were shown to locate on both X and Y chromosomes as detected by FISH with a 18S rDNA probe (Fig. 3d–f). At MII, X and Y chromatids associate forming an XY pseudo-bivalent (Fig. 3f) and segregate reductionally. MI plates are nonradial with X and Y chromosomes distributed among the bivalents (Fig. 3c), whereas MII plates are clearly radial, and XY pseudo-bivalent is located at the center of the ring formed by autosomes (Fig. 3f).

### Cimicidae

***Cimex lectularius* Linnaeus, 1758,** 2n=26+X_1_X_2_Y

not figured

This study confirms that *Cimex lectularius* display 2n=26+X_1_X_2_Y as it was repeatedly reported previously (see Grozeva et al. 2010 and references therein). FISH with a TTAGG probe produced no signals on the chromosome spreads (not shown).

### Lygaeidae s.l.

***Oxycarenus lavaterae* (Fabricius, 1787)**, 2n=14+2m+XY

Fig. 4a, b

**Figure 4a, b.**
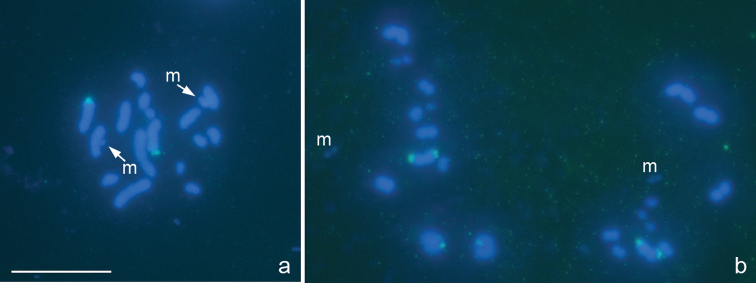
*Oxycarenus lavaterae*, 2n=14+2m+XY. **a** spermatogonial metaphase **b** metaphase I. FISH with a 18S rDNA probe. Bar equals 10 μm.

In accordance with earlier published data (Grozeva 2004), the karyotype of this species includes 18 chromosomes as evidenced by a spermatogonial metaphase (Fig. 4a) and meiotic MI with 8 autosomal bivalents and univalent X and Y chromosomes (Fig. 4b). One of the bivalents is very small and negatively heteropycnotic and taken as a pair of m-chromosomes described earlier in six other species of the subfamily Oxycareninae (Grozeva 1995). The m-chromosomes are likewise well recognized at the spermatogonial metaphase (Fig. 4a). The 18S rDNAsignals could be easily seen on the second largest pair of autosomes (Fig. 4a, b).

### Pyrrocoridae

***Pyrrhocoris apterus* (Linnaeus, 1758),** 2n=22+X

Fig. 5a–f

**Figure 5a–f.**
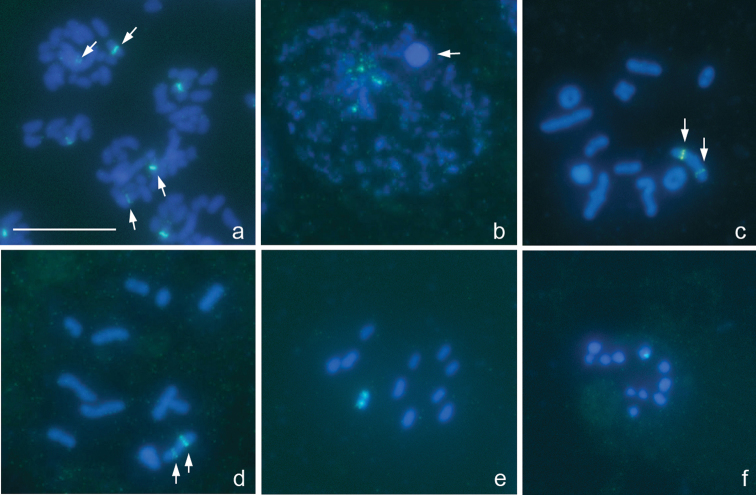
*Pyrrhocoris apterus*, 2n=22+X. **a** spermatogonial prometaphase **b** early prophase (arrowed is the sex chromosome body) **c, d** prometaphase I **e** metaphase II **f** telophase II. FISH with an 18S rDNA probe. Arrowed are 18S rDNA clusters. Bar equals 10 μm.

In accordance with previously published data (Henking 1891, Ueshima 1979, Sahara et al. 1999), the species displays 2n=22+X in males as indicated by our observations of different stages of meiosis (Fig. 5c–f). FISH with an 18S rDNAprobe produced clear interstitial signals on every homologue of a larger autosomal bivalent best demonstrated in Figures 5c and 5d. Figure 5b (meiotic prophase) shows that signals are present on autosomes and absent on a sex chromosome body (arrowed). We call attention to difference in signal strength between the homologues (Fig. 5a, c, d), which is most likely caused by difference in 18S rRNAgene copy number.

### Pentatomidae

***Eurydema oleracea* (Linnaeus, 1758)**, 2n=12+XY

Fig. 6a–f

**Figure 6a–f.**
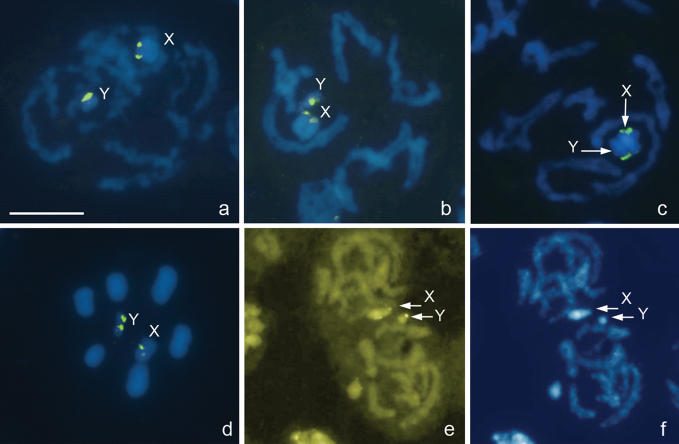
*Eurydema oleracea*, 2n=12+XY. **a-c, e, f** different prophase stages **d** metaphase I. FISH with a 18S rDNA probe **a-d** and CMA_3_ /DAPI **e/f-**staining. Bar equals 10 μm.

This study confirms that *Eurydema oleracea* has 2n=12+XY as previously reported by other researchers (Schachow 1932, Geitler 1939, Xavier and Da 1945). At different prophase stages and at MI (Fig. 6a–d), 6 autosomal bivalents and two univalent sex chromosomes, X and Y, were observed. The chromosomes are fairly large as compared with those in the above mentioned multichromosomal species. In *Eurydema oleracea*, the X chromosome is medium-sized whereas the Y chromosome is the smallest in the set; autosomal bilvalents constitute a decreasing size raw. FISH with a (TTAGG)_n_ probe did not reveal positive signals on chromosomal spreads. Clear 18S rDNAsignals were evident on both sex chromosomes (Fig. 6a–d). Results of fluorochrome staining were consistence with the FISH evidence since CMA_3_-positive/DAPI-negative regions were observed on the sex chromosomes confirming thus the presence here the ribosomal loci (Fig. 6e, f). In meiosis, both MI and MII plates were radial with univalent sex chromosomes at MI (Fig. 6d) and an X and Y pseudo-bivalent at MII (not shown) being located at the centre of the ring formed by autosomes. A number of MI plates demonstrated the deviations from the radiality with some of the autosomal bivalents lying at the center of the ring or one of sex chromosomes lying outside the ring.

***Graphosoma lineatum* (Linnaeus, 1758)**, 2n=12+XY

Fig. 7a–d

**Figure 7a–d.**
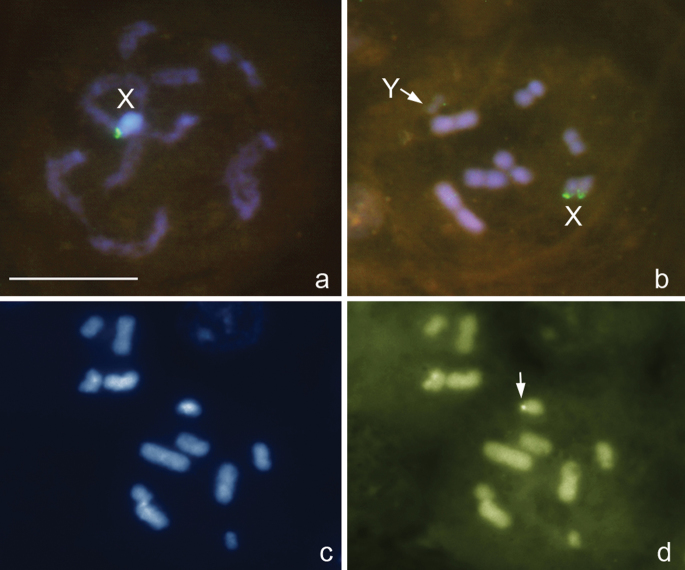
*Graphosoma lineatum*, 2n=12+XY. **a** prophase stage **b–d** metaphase I. FISH with 18S rDNAand (TTAGG)_n_ probes **a, b** and DAPI **c** and CMA_3 _**d**-staining. Arrowed **d** is a CMA_3_-positive signal on the X. Bar equals 10 μm.

The karyotype of 2n=12+XY discovered here in *Graphosoma lineatum* is in accordance with that published previously for other populations of this species (Geitler 1939, Xavier and Da 1945). The karyotype closely parallels that in *Eurydema oleracea*. The chromosomes are fairly large and noticeably larger as compared with the multichromosomal species. At MI, there are 6 bivalents and the univalent X and Y chromosomes, the X chromosome being medium-sized and the Y the smallest chromosome in the set; autosomal bivalents constitute a decreasing size raw (Fig. 7a–d). FISH with a (TTAGG)_n_ probe did not reveal positive signals on the chromosomal spreads. Ribosomal DNA cistrons were found to locate at the terminal position on the X as detected by FISH with an 18S rDNAprobe (Fig. 7a, b) and DNA binding fluorochromes which revealed DAPI-dull/CMA_3_-bright bands on the X (Fig. 7c, d).

### Dot-blot hybridization analysis

Dot-blot hybridization of the genomic DNA from *Cimex lectularius*, *Nabis* sp. and *Oxycarenus lavaterae* was performed using seven telomeric probes, ciliate (TTTTGGGG)_n_ and (TTGGGG)_n_, nematode (TTAGGC)_n_, insect (TTAGG)_n_, shrimp (TAACC)_n_, vertebrate (TTAGGG)_n_ and plant (TTTAGGG)_n_. All the experiments provided no hybridizing bands clearly suggesting some other molecular composition of telomeres in true bugs.

## Discussion

### Standard chromosomal complements

We studied standard chromosomal complements of eight species from 6 genera and 5 families of the true bug infraorders Cimicomorpha (*Deraeocoris rubber*, *Deraeocoris rutilus*, *Megaloceroea recticornis*, *Cimex lectularius*) and Pentatomomorpha (*Oxycarenus lavaterae***,** *Pyrrhocoris apterus*, *Eurydema oleracea*, *Graphosoma lineatum*). Our study confirms the previously published information (see Results and Table 3 for the references) that *Cimex lectularius* (Cimicidae) displays 2n=26+X_1_X_2_Y; *Pyrrhocoris apterus* (Pyrrhocoridae) – 2n=22+X; *Oxycarenus lavaterae* (Ligaeidae) – 2n=14+2m+XY; *Eurydema oleracea*and *Graphosoma lineatum* (Pentatomidae) – 2n=12+XY. On the other hand, Leston (1957) recorded *Megaloceroea recticornis* (Miridae) in England as having 2n=32+XY; however this count was not corroborated by our observations of this species. The karyotype of *Megaloceroea recticornis* in Bulgaria, as revealed in our work, is 2n=30+XY. We can not explain this incompatibility, especially as Leston provided neither photograph nor drawing of the chromosomal complement. It should be mentioned here that 2n=32+XY is the first whereas 2n=30+XY the second commonest karyotype in the Miridae (Kuznetsova et al. 2011). The chromosomal complements of *Deraeocoris rutilus* and *Deraeocoris ruber* were studied herein for the first time. These species were found to agree with one another in a karyotype of 2n=32+XY, with a pair of m-chromosomes among autosomes, and the karyotype formula is hence determined as 2n=30+2m+XY. A pair of chromosomes (the autosomes) known as m-chromosomes has been described in karyotypes of many bug species (Ueshima 1979). These chromosomes are typically extremely small, negatively heteropycnotic and behave differently as compared to autosomes and sex chromosomes during meiosis. However their origin and significance in genomes remain still obscure. The presence or absence of m-chromosomes seems to represent a fairly stable character at higher taxonomic levels in the Heteroptera (Ueshima 1979). Until the present time, m-chromosomes have been discovered in as few as two Miridae species, *Capsus ater* (Linnaeus, 1758) and *Dicyphus digitalidis* Josifov, 1958 (Nokkala and Nokkala 1986, Grozeva and Simov 2008a), even though dozens Miridae species were studied in respect to karyotypes (see review: Kuznetsova et al. 2011). Thus, *Deraeocoris rutilus* and *Deraeocoris ruber* from our study increased the total number of mirid species with m-chromosomes to four. It is worthy of note that m-chromosomes were not described in the ten previously studied representatives of the genus *Deraeocoris* Kirschbaum, 1856 which species were shown to have 2n=32+XY as well (see Ueshima 1979). In some cases m-chromosomes might have been overlooked due to their too small size and negative heteropycnosis in meiosis (Kuznetsova et al. 2011). It remains to be added here that the species studied in this work display holokinetic chromosomes which lack primary constrictions (the centromeres) as in all other Heteroptera (Ueshima 1979).

**Table 3.** Chromosomal complements and 18S DNA locations in the species studied

**Infraorder, family, and species**	**2n** (♂)	**Karyotype formula**	**18S rDN*A* location**	**Published data on karyotype**
**Cimicomorpha**				
**Miridae**				
*Deraeocoris ruber*	34	2n=30+2m+XY	X and Y chromosomes	Absent
*Deraeocoris rutilus*	34	2n=30+2m+XY	X chromosome	Absent
*Megaloceroea recticornis*	32	2n=30+XY	X and Y chromosomes	2n=32+XY (Leston 1957)
**Cimicidae**				
*Cimex lectularius*	29	2n=26+X_1_X_2_Y	X_1_ and Y chromosomes*	2n=26+X_1_X_2_Y / (Grozeva et al. 2010 and references therein)
**Pentatomomorpha**				
**Ligaeidae**				
*Oxycarenus lavaterae*	18	2n=14+2m+XY /	A pair of larger autosomes	2n=14+2m+XY / (Grozeva 2004)
**Phyrrocoridae**				
*Pyrrhocoris apterus*	23	2n=22+X	A pair of larger autosomes	2n=22+XX/X0 (Henking 1891, Ueshima 1979, and references therein; Sahara et al. 1999)
**Pentatomidae**				
*Eurydema oleracea*	14	2n = 12+XY	X and Y chromosomes	2n=12+XY (Schachow 1932, Geitler 1939, Xavier and Da 1945)
*Graphosoma lineatum*	14	2n = 12+XY	X chromosome	2n=12+XY (Geitler 1939, Xavier and Da 1945).

*Data from Grozeva et al. 2010

### Male meiosis

In all the seven species studied in this work, the first meiotic division is reductional for the autosomes and equational for the sex chromosomes, and vice versa – the second division is equational for the autosomes and reductional for the sex chromosomes. Such a behavior of sex chromosomes in male meiosis, or “post-reduction”, as it is called, represents one of the unique cytogenetic characters of the Heteroptera being inherent in most bug species (Ueshima 1979). The species studied herein, all except Miridae species, showed the orthodox chiasmate meiosis in males with only one chiasma per bivalent, the meiotic pattern characteristic of holokinetic chromosomes (Halkka 1964, Nokkala et al. 2004). In common with several Miridae species studied so far in this respect ((Nokkala and Nokkala 1986; Grozeva et al. 2006, 2007; Grozeva and Simov 2008a, b), the three mirid species from our work, *Deraeocoris ruber*, *Deraeocoris rutilus* and *Megaloceroea recticornis*, were found to have achiasmate meiosis of a collochore type (best exemplified by *Megaloceroea recticornis*). In meiosis of this type, diplotene and diakinesis stages are absent, and no chiasmata are formed between homologous which are however connected with each other, generally only at one site, by thread-like structures, the so-called collochores. The collochores have the function to hold homologous chromosomes together in the absence of chiasmata, and hence ensure their proper orientation and regular segregation at anaphase I.

One of the distinctive properties of the true bug meiosis is a specific spatial arrangement of metaphase plates known as radial ones. Either at both metaphases, MI and MII, or at only one of those, the autosomes (either as bivalents at MI or as univalents at MII) form a ring with the sex chromosomes (either as univalents at MI or as a pseudo-bivalent at MII) lying in its center (Ueshima 1979). In two species studied here on this point, different patterns were observed. The mirid species *Megaloceroea recticornis* displayed MI plates nonradial with X and Y chromosomes distributed among the bivalents, and MII plates clearly radial with XY pseudo-bivalent located at the center of the ring formed by autosomes. Based on our observations of *Eurydema oleracea*, in this pentatomid species both MI and MII plates are radial. We emphasize however that MII plates in this species were more stable in this pattern compared to MI plates, which sometimes demonstrated the deviations from the radiality with some of the autosomal bivalents also lying at the center of the ring or one of sex chromosomes lying outside the ring. The differences between MI and MII in regard to their radial arrangement observed in *Eurydema oleracea* are in agreement with the available data on species from other bug families, including the Pentatomidae (Rebagliati et al. 2003). In another pentatomid species, *Graphosoma lineatum*, the first metaphase was nonradial, however there was no MII plates to be analyzed.

### Chromosomal location of 18S rDNA clusters

The nucleolus represents a subnuclear compartment of eukaryotic cells in which the synthesis of ribosomal RNA (rRNA) and formation of ribosomes take place (Busch and Smetana 1970). Nucleolar organizer regions (NORs) are usually detected in insects by silver nitrate (AgNO_3_) and GC-specific fluorochrome (most commonly by CMA_3_) staining. However silver treatment stains only active NORs (Hubbell 1985) being therefore inadequate to the study of NOR location onto chromosomes. In contrast, fluorescence *in situ* hybridization (FISH) with rDNA probes directly detects the location of ribosomal RNA genes, regardless of their activity. In eukaryotes, *5S* and *18S* ribosomal genes (rDNA) are organized into two multigenic families, namely the major rDNA family formed by the *18S*, *5.8S*, and *28S* genes and the minor one composed of *5S* genes (Long and David 1980). Chromosomal mapping of genes is important for identification of chromosomes, which is especially difficult in groups of organisms with holokinetic chromosomes.

In Heteroptera, physical location of genes remains very poorly sampled (and the data available concern only ribosomal genes), mainly with sporadic sampling of a few select species. Out of more than 40,000 described species (Weirauch and Schuh 2011), approximately 1600 species have been subjected to cytogenetic analysis (Papeschi and Bressa 2006). Among those, only 22 species (11 genera) belonging to the families Reduviidae (Severi-Aguiar et al. 2005, 2006, Morielle-Souza and Azeredo-Oliveira 2007, Bardella et al. 2010, Panzera et al. 2010, Poggio et al. 2011), Cimicidae (Grozeva et al. 2010), Coreidae (Papeschi et al. 2003, Cattani et al. 2004, Bressa et al. 2008), Belostomatidae (Papeschi and Bressa 2006), Pentatomidae (Papeschi et al. 2003), and Pyrrhocoridae (Bressa et al. 2009) have been investigated in respect to FISH rDNA location.

In this study we have characterized the chromosomal locations of 18S RNA genes in seven species from six genera of the families Miridae (*Deraeocoris ruber*, *Deraeocoris rutilus*, *Megaloceroea recticornis*), Lygaeidae (*Oxycarenus lavaterae*), Pyrrhocoridae (*Pyrrhocoris apterus*), and Pentatomidae (*Eurydema oleracea*, *Graphosoma lineatum*). Data on all the species (as well as those on the whole families Miridae and Lygaeidae) were obtained for the first time bringing thus the total number of the species and genera studied to 30 and 18, respectively. The species were shown to exhibit different patterns of rDNA location. Some of the species showed their ribosomic cistrons located on sex chromosome, either on the X (*Deraeocoris rutilus*) or on both X and Y(*Deraeocoris ruber*, *Megaloceroea recticornis*, *Eurydema oleracea*, *Graphosoma lineatum*) whereas in other species (*Oxycarenus lavaterae*, *Pyrrhocoris apterus*) they were located on a pair of autosomes.

The location of 18S rDNAappeared different within the taxa, in which rDNA sequences were mapped in more than one species as in the mirid genus *Deraeocoris*, where *Deraeocoris rutilus* displayed rDNA clusters concentrated on the X, but *Deraeocoris ruber* on both X and Y chromosomes. These findings give no way of any inferences especially as a wide variation of chromosomal location for the major rDNA has been observed in different bug taxa, including variations between the co-generic species. For example, the genus *Triatoma* Laporte, 1832 from the subfamily Triatominae (Reduviidae), which is one of the most studied bug groups, clearly shows the interspecific variation (Severi-Aguiar et al. 2005, 2006, Morielle-Souza and Azeredo-Oliveira 2007, Bardella et al. 2010, Poggio et al. 2011) while sometimes even intraspecific variation (Panzera et al. 2010) for the major rDNA harboring either on the sex chromosomes (X and/or Y), or the autosomes or on both. This variability is suggested to be due to the chromosomal exchanges between the autosomes and sex chromosomes during the speciation of the Triatominae (Panzera et al. 2010).

### The telomere repeat sequence

The pentanucleotide sequence (TTAGG)_n_ is known as the commonest and most likely an ancestral DNA motif of insect telomeres (Sahara et al. 1999, Frydrychová et al. 2004). However this motif was lost during the evolution of several groups being secondarily replaced with another motif (yet unknown) or the alternative telomerase-independent mechanisms of telomere maintenance (Frydrychová et al. 2004, Lukhtanov and Kuznetsova 2010). The true bugs are considered as one of the insect higher taxa in which (TTAGG)_n_ is absent, however the data available concerned so far the two species only, *Halyomorpha halys* (Stål, 1855) (Pentatomidae) studied using Southern hybridization (Okazaki et al. 1993: as *Halyomorpha mista* (Uhler, 1860)) and *Pyrrhocoris apterus* (Pyrrhocoridae) subjected both to Southern hybridization and FISH (Sahara et al. 1999). Comparative analysis of the occurrence of (TTAGG)_n_ in various groups of insects has showed that this motif is evolutionarily stable, and, having once appeared during evolution, marks taxa and phylogenetic branches of high rank. It is known however that in some groups, such as the orders Coleoptera and Neuroptera, both TTAGG-positive and TTAGG-negative species are encountered (Frydrychová et al. 2004 and references therein). By using FISH we studied the occurrence of (TTAGG)_n_ telomere repeat in five species: *Deraeocoris ruber* and *Megaloceroea recticornis* (Miridae), *Cimex lectularius* (Cimicidae), *Eurydema oleracea* and *Graphosoma lineatum* (Pentatomidae). All these species were shown to lack the insect consensus sequence. Although in both mirid species a number of prominent hybridization signals could be seen on separate chromosomes, these signals most likely did not indicate the telomeres. The presence of signals suggests a sequence related to TTAGG but it seems to have no target specificity in the bug chromosomes.

The absence of (TTAGG)_n_ telomeric repeat in the phylogenetically distant groups within the Heteroptera strengthens thus the view (Frydrychová et al. 2004) that it was lost in early evolution of this group of insects.

Dot-blot hybridization of the genomic DNA from bug species with seven types of telomeric probes, ciliate (TTTTGGGG)_n _and (TTGGGG)_n_, nematode (TTAGGC)_n_, insect (TTAGG)_n_, shrimp (TAACC)_n_, vertebrate (TTAGGG)n and plant (TTTAGGG)_n_, yielded negative results and did not provide hence any answer of the question which is the telomere repeat sequence in bug chromosomes.
